# Endangered status and threatened population ecological factors in *Salvia daiguii*, an endemic species from Hunan, China

**DOI:** 10.1002/ece3.11629

**Published:** 2024-06-24

**Authors:** Han‐Wen Xiao, Qing‐Shan Liu, Yan‐Bo Huang, Yan Li, Yu‐Kun Wei, Ru‐Nan Tian

**Affiliations:** ^1^ College of Landscape Architecture Nanjing Forestry University Nanjing China; ^2^ Shanghai Chenshan Botanical Garden Shanghai China; ^3^ Shanghai Botanical Garden Shanghai China

**Keywords:** endangered species conservation, niche, PSESP, redundancy analysis, *Salvia*, soil physicochemical properties

## Abstract

Many species of *Salvia* have excellent ornamental, culinary, and medicinal values. *Salvia daiguii*, is an ornamental and highly medicinal perennial herb endemic to the prefecture‐level city of Zhangjiajie in Hunan Province, China, with a narrow geographical distribution. However, currently, it has only been assessed as a Critically Endangered species according to the IUCN classification criteria, but its conservation has not yet been studied. This study investigated the distribution and niche characteristics of *S. daiguii*, and compared the differences in growth, flowering characteristics, and soil nutrients between the wild and ex situ populations. We also analyzed the effects of soil nutrients on plant growth and flowering characteristics. During the survey, we found 274 individuals on a rock approximately 200 m from ZEFR1. Nevertheless, *S. daiguii* were still restricted in three populations, TNFP, TGM, and ZEFR in Zhangjiajie City, with a total of about 500 plants and less than 250 mature individuals. Our results show that aspects such as adverse environmental conditions, low seedling renewal rate, a lack of soil nutrients, and competition for the characteristic niche of this and other dominant plants in the natural community are the main ecological factors affecting the growth, flowering, and geographic distribution of *S. daiguii*. Based on the results of field surveys, we recommend that (1) *S. daiguii* be classified as Critically Endangered C2b and China's List of Plant Species with Extremely Small Populations. (2) Comprehensive conservation strategies were developed, such as the establishment of nature reserves, reintroduction, public education, and institutional development to provide management recommendations related to the conservation of *S. daiguii* and other endangered plants.

## Graphical abstract

Currently, *Salvia daiguii* is distributed in three populations, with total of about 500 plants and less than 250 mature individuals. Habitat OM and pH are indicators that significantly affect the growth and flowering of *S. daiguii*. Competition for niche characteristics of dominant plants in the community affects the growth and dispersal of *S. daiguii*. Finally, a comprehensive conservation strategies were developed, as the establishment of nature reserves, reintroduction, and public education, etc.

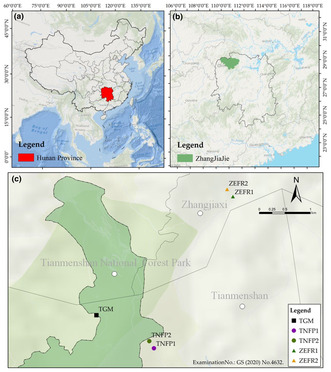

## 1 INTRODUCTION

The Intergovernmental Panel on Climate Change (IPCC) and the Intergovernmental Science‐policy Platform on Biodiversity and Ecosystem Services (IPBES) (Pörtner et al., 2021, IPCC‐IPBES scientific outcome) report that one million species on Earth are threatened with extinction and that many plants are predicted to become extinct in the coming decades. Qin et al. (2017) reported that one‐tenth of the 36,000 species of higher plants in China are threatened with extinction. With increasing socioeconomic development and intensifying human activities, several stress factors impacting biodiversity are predicted to result in major dangers to the survival of many wild plants or even pose the threat of extinction (Bellard et al., 2012; Butchart et al., 2010; Tilman et al., 1994; Xu et al., 2022). These threats include the effects of habitat fragmentation, human resource consumption and excessive exploitation of resources, habitat degradation, non‐native invasive species, nitrogen pollution, limited natural reproduction, and the effects of climate change (Bellard et al., 2012; Butchart et al., 2010; Cowie et al., 2022; Johnson et al., 2017). Plant resources are the basis for human survival and development and can help to guarantee the maintenance of sustainable human social and economic development (Xu et al., 2022). The rapid extinction of endangered plants may lead to the destruction of the global ecosystem, which will seriously threaten the survival and development of humans (Wen & Chen, 2022; Xu et al., 2022). Therefore, understanding the causes of plant extinction and formulating reasonable conservation strategies are the major challenges facing humans currently.

Information on the distribution of plants in the field, including location and range of distribution, habitat, population size, abundance, plant growth state, and soil nutrition, is significant in determining the threat level to plants and related conservation priorities (Cogoni et al., 2021; Dabré et al., 2023; Krebs, 2015; Robiansyah et al., 2021; Wang et al., 2016; Xu & Zang, 2022; Zhang et al., 2022). Moreover, the goal of biodiversity conservation is to conserve species and the habitats in which they occur (Essl et al., 2009); however, habitat loss may cause a significant threat including a decline in biodiversity, especially for an endangered species with a narrow geographical distribution (Paglia et al., 2022; Xu et al., 2021). Therefore, knowing the distribution and habitats of endangered plants can help researchers to better understand the endangered status of plants, so that they can conserve endangered species more effectively.

The concept of a niche refers to the interrelationships among individuals of a species within a population and between the species and the environment a population inhabits including the concepts of species importance values, niche width, and niche overlap (Grinnell, 1917; Hurlbert, 1978; Leibold & Geddes, 2005; Spies et al., 1990). These niche characteristics can reflect the species composition, structure, status, and competitive adaptations in a population (Komonen & Kotiaho, 2021; Peterson, 2011; Suárez‐Mota & Villaseor, 2020; Wiens et al., 2010). Therefore, the analysis of niche characteristics of endangered plants helps researchers comprehend the status and the state of resource use of these species in a community, which is of great scientific significance for clarifying the causes of threats to a plant species and formulating corresponding conservation measures.

The genus *Salvia* includes about 1000 species, making the genus widely distributed worldwide; various species are widely used in ornamental, culinary, and medicinal fields due to their beautiful plant forms, diverse flower shapes and colors, and richness in many aromatic substances and medicinal ingredients (Hu et al., 2023; Xiao et al., 2021; Xiao, Huang, et al., 2023; Xiao, Huang, Wang, et al., 2022; Xiao, Liu, et al., 2023; Zheng et al., 2013). Forty‐five of these are classified as threatened species by the International Union for Conservation of Nature (IUCN; https://www.iucnredlist.org/). According to Qin et al. (2017), the Chinese List of Threatened Species of Higher Plants, there are just four species of sage that are considered threatened in China. Nevertheless, the conservation of these very valuable ornamental and medicinal endangered species is frequently overlooked.

**FIGURE 1:**
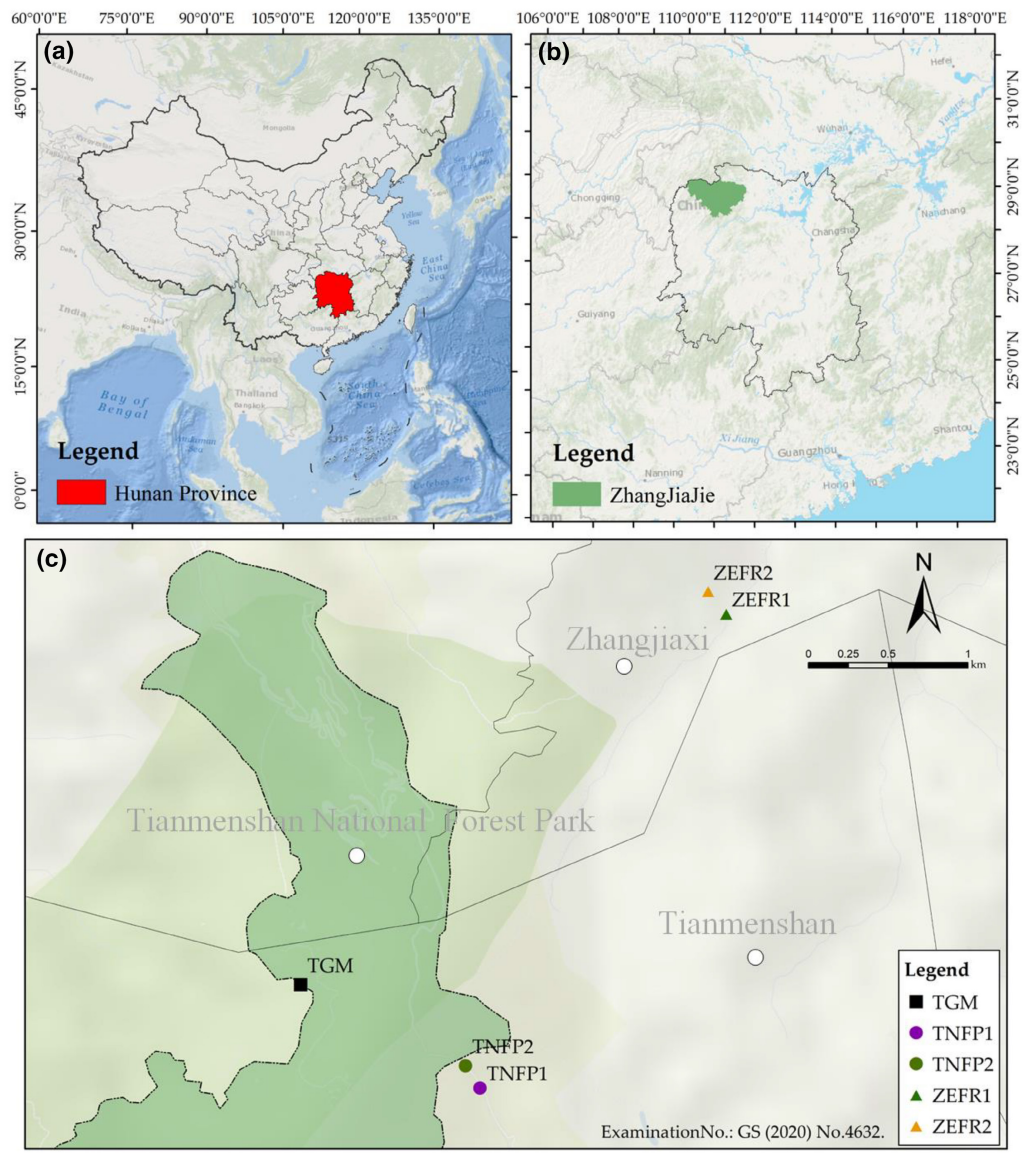
Distribution of *Salvia daiguii*: (a) red indicates the Hunan Province; (b) green indicates Zhangjiajie City; (c) the distribution of *S. daiguii* in Zhangjiajie City. TGM, Tianmenshan Guigu Moat; TNFP, Tianmenshan National Forest Park; ZEFR, Zhangjiaxi Ecological Forest Reserve.

*Salvia daiguii* was first discovered in 2011 in Wulingyuan, Zhangjiajie City, Hunan Province, China. In 2019, *S. daiguii* was described as a new species after morphological comparisons revealed that it was clearly distinct from *Salvia cavaleriei*. The new species is known from two sites, each with no more than 200 individuals at that time (Figure 1, TNFP 1 and TGM), and the type specimen was stored in the Chenshan Herbarium of Shanghai Chenshan Botanical Garden (CSH, Wei et al., 2019). Physiological tests indicated that *S. daiguii* is tolerant to varying degrees of stress such as high temperature, high humidity, drought, salinity, and acid rain (Chen et al., 2023). This species can provide excellent material to be used as a cultivated ornamental plant and as an artificial hybrid for producing new species (Chang et al., 2020; Lin et al., 2024). Also, its high content of caffeic acid, salicylic acid B, and rosmarinic acid in the roots provide high medicinal value (Wang, 2020). However, currently, it has only been assessed as a Critically Endangered species according to the IUCN classification criteria (Wei et al., 2019; Xiao, Huang, & Wei, 2022), and its conservation has not yet been studied.

The present study reinvestigated the distribution and habitat of *S. daiguii*, studied its niche characteristics, and compared the plant growth, flowering characteristics, and soil nutrients of plants in ex situ conservation in SCBG with those growing in the wild populations. We also analyzed the effects of soil physicochemical properties on plant growth and flowering. We tried to answer the following questions: (1) What is the distribution of *S. daiguii* and are the populations declining? Is it facing extinction? (2) How do the plant growth, flowering characteristics, and soil nutrients of the *S. daiguii* field populations compare with those of plants in ex situ conservation in SCBG? Do soil nutrients affect the growth and flowering of these plants? (3) What are the niche characteristics of *S. daiguii* in the population? On this basis, we developed comprehensive conservation recommendations to inform the recovery and sustainable use of *S. daiguii* populations.

## 2 MATERIALS AND METHODS

### 2.1 Study sites and species

The field study was conducted in Yongding District, Zhangjiajie City, Hunan Province, China. The study site is a complete syncline tectonic unit and belongs to the quartz sandstone peak forest landform (Yan, 2003). The region has a humid subtropical monsoon climate with an average annual precipitation of 1520.6 mm and an average annual temperature of 16.6°C (Yan, 2003). Currently, *S. daiguii* has been successfully conserved ex situ in SCBG by in vitro, division, and sowing methods (Xiao, Huang, & Wei, 2022), which has provided us with a basis for further analysis of the effects of soil nutrients on plant growth and flowering characteristics. For the present study, *S. daiguii* plants were planted in a mixture of peat soil, humus, and vermiculite at a ratio of 3:3:1. To prevent soil caking and to ensure adequate soil nutrients were available, the soil was replaced once a year. Plant growth, flowering indicators, and soil nutrient measurements for ex situ conservation were performed at SCBG.

Mature *S. daiguii* plants grow to a height of 13–21 cm, with white flowers arranged in densely verticillasters forming 8–15 cm racemes. *Salvia daiguii* has four ovules per flower and produces a small amount of nectar at the base of the corolla tube. Previous studies found that *Apis cerana* is the primary pollinator of *S. daiguii*, but its curved style allows the nectar thief *Macroglossum bombylan*s to become a secondary pollinator (Xiao, Liu, et al., 2023).

### 2.2 Distribution and the habitat survey of *S. daiguii*

Researchers from the Shanghai National Labiatae Germplasm Resource Bank over 25 provinces and cities extensively surveyed between 2011 and 2021, and *S. daiguii* was found at only three sites in Zhangjiajie City (Wei et al., 2019; Xiao, Liu, et al., 2023; see Figure 1, TNFP [TNFP 1 and 2], ZEFR 1, and TGM). To investigate the distribution of *S. daiguii*, we re‐surveyed the three previous locations. We also consulted relevant experts and field guides, and conducted field surveys in Zhangjiajie City and five surrounding counties (Cili, Sangzhi, Yongshun, Baojing, and Guzhang, respectively) within 4000 km^2^ from 2021 to 2022. The location (e.g., elevation, latitude, and longitude) of *S. daiguii* in the wild was recorded by GPS, and habitat and plant growth characteristics were photographed with a Nikon D90. Subsequently, the number of plants per population (including the total number of plants, the number of flowering plants, the number of young to adult plants, and the number of seedlings) was counted.

### 2.3 Growth and flowering characteristics of *S. daiguii*

To compare the differences in growth and flowering characteristics of *S. daiguii* in different populations. According to the distribution range and numbers of plants in each population of *S. daiguii*, in June–July 2022, 10, 7, 10, and 5 individuals were randomly selected from the ex situ populations conserved at SCBG and from the wild populations of TNFP, Zhangjiaxi Ecological Forest Reserve (ZEFR), and Tianmenshan Guigu Moat (TGM), respectively. We counted the growth and flowering indicators of *S. daiguii*, including four growth indices of plant height (PHE), crown diameter (CD), number of tillers per plant (NT), and number of leaves per plant (NL). In addition, seven flowering indices were calculated including numbers of inflorescences per individual plant (NI), branches per inflorescence (NB), verticillasters per branch (NV), flowers per verticillaster (NF), blooming flowers per verticillaster on the same day (NBF), blooming flower per individual plant per day (NBFP), and flowers per individual plant (NFP).

### 2.4 Physicochemical properties of *S. daiguii* soil

To compare the soil nutrient status of *S. daiguii* in different areas of distribution. We collected 0–20 cm of soil in the root zone of *S. daiguii* at the following three populations: from the ex situ conservation site at SCBG and the wild populations at TNFP and ZEFR, in June–July 2022. Three samples were taken from each of the three populations. Because the TGM population occurs on roadside cliffs of the core scenic area of Tianmenshan National Forest Park, soil was only sampled from two buffer areas of TNFP and ZEFR sites to minimize damage to the scenic area. These soil samples were air‐dried in the laboratory to remove impurities and stones and then sieved through a 2‐mm sieve before determining the soil's physicochemical properties. Soil potential hydrogen (pH) was determined using 0.01 mol/L CaCl (soil/water extracts 1:2.5). Soil electrical conductivity (EC) was measured with 250 mL of distilled water extract (soil/distilled water 1:5). Soil organic matter (OM) was determined with potassium dichromate (K_2_Cr_2_O_7_). Soil nitrogen (N), phosphorus (P), potassium (K), available nitrogen (AN), available phosphorus (AP), available potassium (AK), boron (B), and sulfur (S) were determined with 1 M HCl solution with an extractant volume ratio of 1:5. Soil cation exchange capacity (CEC) was detected with 1 mol/L ammonium acetate (CH_3_COONH_4_, volume ratio of 1:30). The soil moisture (SM) content was determined by the drying method, i.e, the weight of wet soil minus the weight of the soil after drying divided by the wet soil weight. All of the above‐mentioned soil physicochemical properties were measured according to the Methods in the National Standards of China (Liu, 1996), and all soil tests were repeated three times.

### 2.5 Niche characteristics of *S. daiguii*

During June–July 2022, based on the sites of *S. daiguii* occurrence and the habitat type field surveyed, a total of four 5 m × 10 m sample plots (two for each population) were set up in two populations of *S. daiguii* (TNFP and ZEFR). The ecological factors of plants within each sample plot to calculate important values, such as the number of plants, PHE, and percent cover were measured. The niche characteristics of *S. daiguii* were quantified using the Levins (Levins, 1968; *B*_*i*_) and Shannon (Shannon & Weaver, 1949; *B*_*s*_) niche width and the Pianka niche overlap index (Pianka, 1973; *O*). The calculation method is as follows:

(1) $$\text{Importance value}\;\left(\mathrm{IV}\right)=\left(\mathrm{RA}+\mathrm{RH}+\mathrm{RC}\right)/3,$$

(2) $$\text{Levins}\;\left(B_{i}\right)=\frac{1}{\sum_{i=1}^{s}P_{i}^{2}},$$

(3) $$\text{Shannon}–\text{Wiener}\;\left(B_{s}\right)=-\sum_{i=1}^{s}p_{i}\ln\left(P_{i}\right),$$

(4) $$\text{Niche overlap}\;\left(O\right)=\frac{\sum_{k-1}^{s}P_{\mathrm{ik}}P_{\mathrm{jk}}}{\sqrt{\sum_{k-1}^{s}\left(P_{\mathrm{ik}}\right)2\sum_{k-1}^{s}\left(P_{\mathrm{jk}}\right)}2},$$

where RA is the relative abundance, RH is the relative height, and RC is the relative cover; *P*_*i*_ is the proportion of the IV of species in the *i*th to the total IV of the species; *P*_*ik*_ and *P*_*jk*_ is the proportion of the IVs of species *i* and *j* on the *k*th to the total IV of the species; and *S* is the number of quadrats.

### 2.6 Statistical analyses

All statistical analyses were performed using SPSS 22.0 (SPSS, Inc., Chicago, IL, USA). The niche importance value, niche width, and overlap were all calculated in Excel 2019 according to the relevant formulae. Differences in plant growth and flowering characteristics of *S. daiguii* in ex situ conservation and wild populations were analyzed using a generalized linear mixed model (GLLM, Poisson distribution with a log link). We used population location as a fixed factor, plant growth characteristics and flowering characteristics as the dependent variables, and plants as a random factor. A Tukey's multicomparison of one‐way ANOVA was used to analyze the differences in soil physicochemical properties between populations. In addition, we performed redundancy analysis (RDA) using Canoco 5.0 (Microcomputer Power, Ithaca, NY, USA) to explore the effects of soil nutrients on plant growth and flowering characteristics between populations, and indicators of soil physicochemical properties as explanatory variables.

## 3 RESULTS

### 3.1 Distribution patterns and habitat characteristics of *S. daiguii*

During the 2021–2022 survey, 274 individuals were found on a rock approximately 200 m from ZEFR1 (Figure 1‐ZEFR 2, Table 1). Based on previous and current surveys, *S. daiguii* is still only distributed in three populations, TNFP (TNFP 1 and TNFP 2), TGM, and ZEFR (1 and 2) (Figure 1).

**TABLE 1:** Numbers of *Salvia daiguii* individuals in five sites in Zhangjiajie City.

	TNFP1			TNFP2		ZEFR1		ZEFR2	TGM			No. of the total as of July 2022
	2019	2021	2022	2021	2022	2021	2022	2022	2019	2021	2022	
No. of plants	<200	16	15	204	173	24	14	274	<200	15	30	506
No. of adult plants	—	0	3 (20%)	63 (30.88%)	65 (37.58%)	5 (20.83%)	3 (21.43%)	36 (13.14%)	—	4 (26.67%)	11 (36.67%)	118 (23.32%)
No. of juvenile plants	—	15 (93.75%)	12 (80%)	122 (59.80%)	90 (52.02%)	19 (79.17%)	11 (78.57%)	233 (85.04%)	—	11 (73.33%)	19 (63.33%)	365 (72.13%)
No. of seedling plants	—	1 (6.25%)	0	19 (9.31%)	18 (10.40%)	0	0	5 (1.83%)	—	0	0	23 (4.55%)

*Note*: Tianmenshan Guigu Moat (TGM); Tianmenshan National Forest Park (TNFP1 and TNFP2); Zhangjiaxi Ecological Forest Reserve (ZEFR1 and ZEFR2). The number of plants in 2019 from Wei et al. (2019).

**FIGURE 2:**
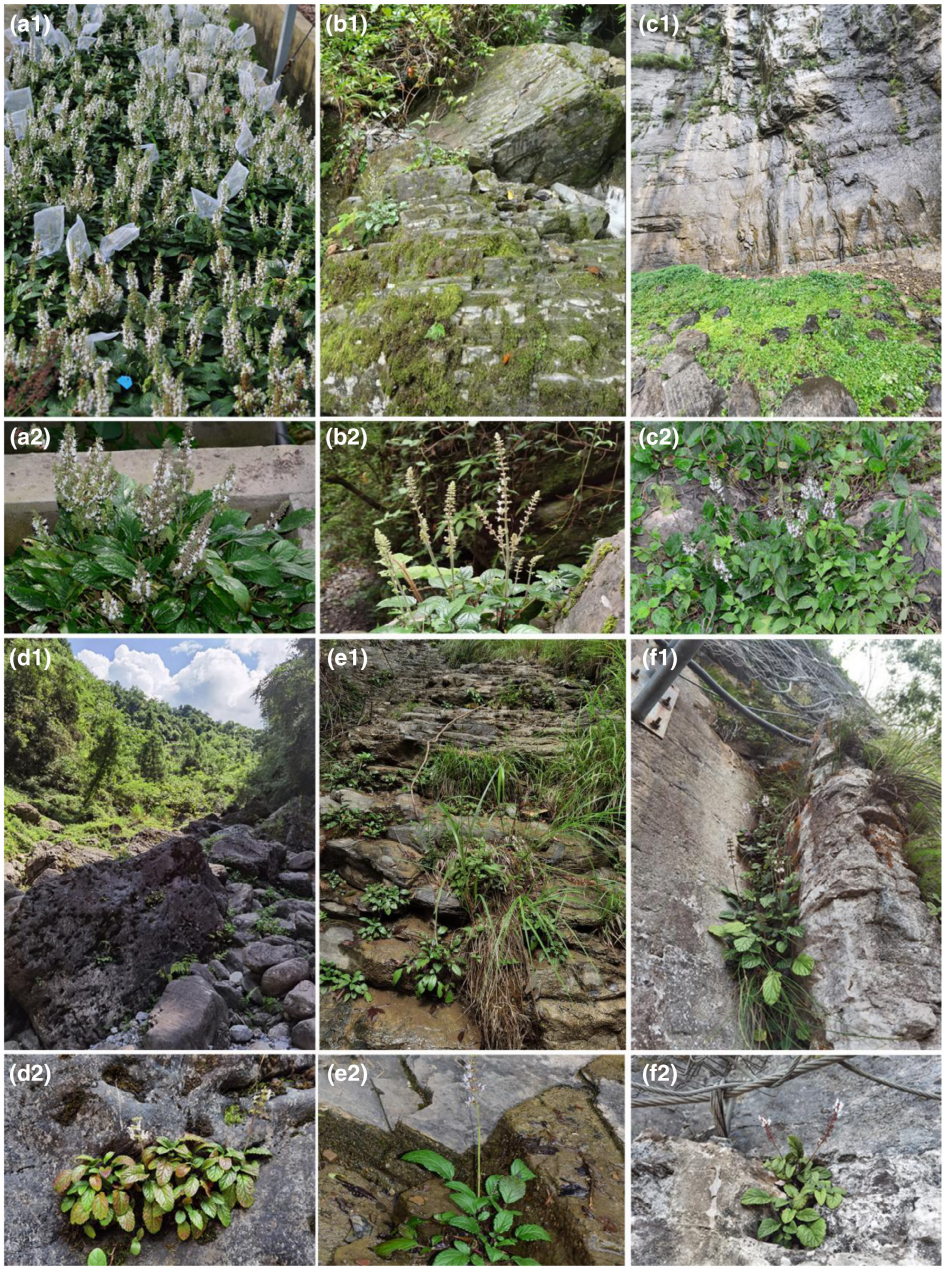
Habitat and plant growth characteristics of *Salvia daiguii* at: (a1, a2) Shanghai Chenshan Botanical Garden (SCBG); (b1, b2) TNFP; (c1, c2) TNFP2; (d1, d2) ZEFR1; (e1, e2) ZEFR2; and (f1, f2) TGM; site acronyms are provided in the legend of Figure 1.

As of July 2022, our surveys found 30 individuals of the TGM population distributed in the crevices of rocks near a tourist trail (Figure 2f). TNFP 1 (Figure 2b) and ZEFR 1 (Figure 2d) had 15 and 14 individuals mainly distributed on rocks in a streambed. The TNFP 2 (Figure 2c) and ZEFR 2 (Figure 2e) sites supported the two most abundant populations found to date, with 173 and 274 individuals mainly distributed on the rocks of a cliff and debris below the cliff. The number of surviving plants at all sites of *S. daiguii* totaled 506 individuals, and the number of mature individuals is less than 250 (Figure 1, Table 1). The distribution population of *S. daiguii* was dominated by young plants (50%–95%) with limited adult plants (0%–38%) and seedling recruitment (0%–10%, Table 1). There was a decrease in the number of plants in all sites compared with the previous survey (Table 1).

### 3.2 Growth and flowering characteristics of different populations of *S. daiguii*

Significant differences were observed in the growth and flowering indicators of individual plants growing in the wild habitats compared with ex situ in SCBG situations (Table 2, Figure 2a1–f2). All growth indicators of *S. daiguii* protected ex situ at SCBG were significantly higher than those of the three populations growing in the wild. At SCBG, all flowering indicators were significantly higher than those of the other three populations growing in the wild, except for the number of verticillasters per branch and the number of blooming flowers per individual plant per day, which were not significantly different from TNFP populations (Table 2). In the TNFP populations, all growth and flowering indicators were significantly higher than those in plants in the ZEFR and TGM populations, except for the number of inflorescences per individual plant and the number of blooming flowers per verticillaster on the same day, which did not differ from the ZEFR population (Table 2). Plants at the TNFP were second only to those in ex situ conservation at SCBG in terms of plant growth and flowering indices. In the TGM population, the growth and flowering indices were not significantly different from those of the ZEFR population, except for the number of inflorescences per plant, which was significantly lower than that of the ZEFR plants (Table 2). The plants of these two wild populations grew shorter and had fewer branches, leaves, inflorescences, and flowers than plants at the ZEFR site. Plant growth and flowering indices in the ZEFR population were significantly lower than those of ex situ plants in SCBG and TNFP populations in the wild (Figure 2a–f, Table 2).

**TABLE 2:** Growth and flowering characteristics of four populations of *Salvia daiguii*.

Plant growth and flowering traits	Sites				*p*‐Value
	SCBG	TNFP	ZEFR	TGM	
Plant height (PHE; cm)	29.25 ± 0.66 a	23.71 ± 2.73 b	13.50 ± 1.28 c	12.40 ± 3.00 c	<.001
Crown diameter (CD; cm)	30.90 ± 1.94 a	22.00 ± 2.69 b	12.15 ± 1.20 c	13.10 ± 2.41 c	<.001
No. of tillers per plant (NT)	15.5 ± 1.07 a	3.86 ± 0.77 b	1.60 ± 0.25 c	1.40 ± 0.22 c	<.001
No. of leaves per plant (NL)	63.00 ± 5.82 a	28.14 ± 4.23 b	14.60 ± 2.00 c	11.60 ± 2.24 c	<.001
No. of inflorescence per individual plant (NI)	12.00 ± 0.62 a	3.57 ± 0.72 b	3.90 ± 1.08 b	1.20 ± 0.18 c	<.001
No. of branches per inflorescence (NB)	7.07 ± 0.28 a	5.16 ± 0.42 b	2.82 ± 0.69 c	1.80 ± 0.33 c	<.001
No. of verticillaster per branch (NV)	10.14 ± 0.13 a	10.22 ± 0.39 a	7.67 ± 0.39 b	8.63 ± 1.07 b	<.001
No. of flowers per verticillaster (NF)	8.54 ± 0.19 a	6.40 ± 0.15 b	5.18 ± 0.12 c	5.12 ± 0.17 c	<.001
No. of blooming flowers per verticillaster on same day (NBF)	4.36 ± 0.14 a	2.72 ± 0.12 b	3.01 ± 0.15 b	2.56 ± 0.25 b	<.001
No. of blooming flowers per individual plant per day (NBFPI)	220.70 ± 26.86 a	46.43 ± 9.21 a	20.00 ± 4.06 b	15.4 ± 1.12 b	<.001
No. of flowers per individual plant (NFP)	1638.40 ± 126.26 a	473.29 ± 86.13 b	153.80 ± 35.27 c	69.80 ± 6.95 c	<.001

*Note*: Different lowercase letters in the table indicate significant differences in growth or flowering indicators between populations (*p* < .05).

### 3.3 Soil physicochemical properties of different populations of *S. daiguii*

The analysis of soil physicochemical properties of different populations showed that all physicochemical property indices in the three populations were significantly different, except for SM content (Table 3). Soil pH in the ex situ plants at SCBG was neutral, while soil pH in the two wild populations was significantly higher than that of those at SCBG, making them slightly alkaline. In general, soil from ex situ conservation plants had higher soil nutrients. However, although SCBG soil K content was lower than ZEFR soil K content, AK was significantly higher in the soil of SCBG than in the two wild populations (Table 3). No significant differences were observed in P and AP between the two wild populations, and all soil nutrient indicators were significantly higher at the TNFP population than at the ZEFR population, except for K and AK, which were significantly lower at the TNFP population (Table 3).

**TABLE 3:** Physicochemical property indices of wild and ex situ soils of *Salvia daiguii*.

Soil physicochemical indices	Sites			*p*‐Value
	SCBG	TNFP	ZEFR	
Potential hydrogen (pH)	7.63 ± 0.02 b	7.98 ± 0.03 a	8.02 ± 0.01 a	<.001
Electrical conductivity (EC; ms/cm)	0.26 ± 0.01 a	0.15 ± 0.003 b	0.09 ± 0.001 c	<.001
Organic matter (OM; g/kg)	118.17 ± 6.42 a	87.49 ± 3.21 b	44.27 ± 2.76 c	<.001
Nitrogen (N; g/kg)	6.25 ± 0.51 a	5.02 ± 0.11 b	3.31 ± 0.18 c	<.001
Phosphorus (P; g/kg)	3.47 ± 0.20 a	0.65 ± 0.02 b	0.48 ± 0.004 b	<.001
Potassium (K; g/kg)	19.19 ± 0.75 b	9.51 ± 0.46 c	35.09 ± 1.17 a	<.001
Available nitrogen (AN; mg/kg)	364.19 ± 14.52 a	291.54 ± 13.27 b	232.68 ± 10.69 c	<.001
Available phosphorus (AP; mg/kg)	373.54 ± 25.38 a	16.89 ± 1.68 b	12.53 ± 0.20 b	<.001
Available potassium (AK; mg/kg)	162.78 ± 3.64 a	99.49 ± 2.63 c	119.89 ± 2.87 b	<.001
Cation exchange capacity (CEC; mg/kg)	16.99 ± 0.90 a	13.48 ± 0.77 b	9.10 ± 1.22 c	<.001
Boron (B; mg/kg)	1.85 ± 0.05 ab	1.95 ± 0.06 a	1.73 ± 0.01 b	.012
Sulfur (S; mg/kg)	51.05 ± 3.37 a	30.98 ± 1.86 b	18.88 ± 1.85 c	<.001
Soil moisture (SM; %)	76.73 ± 7.45 a	56.17 ± 3.89 a	74.47 ± 11.47 a	.350

*Note*: Different lowercase letters in the table indicate statistically significant differences in soil physicochemical property indicators between populations (*p* < .05).

### 3.4 Effect of soil physicochemical properties on plant growth and flowering

**FIGURE 3:**
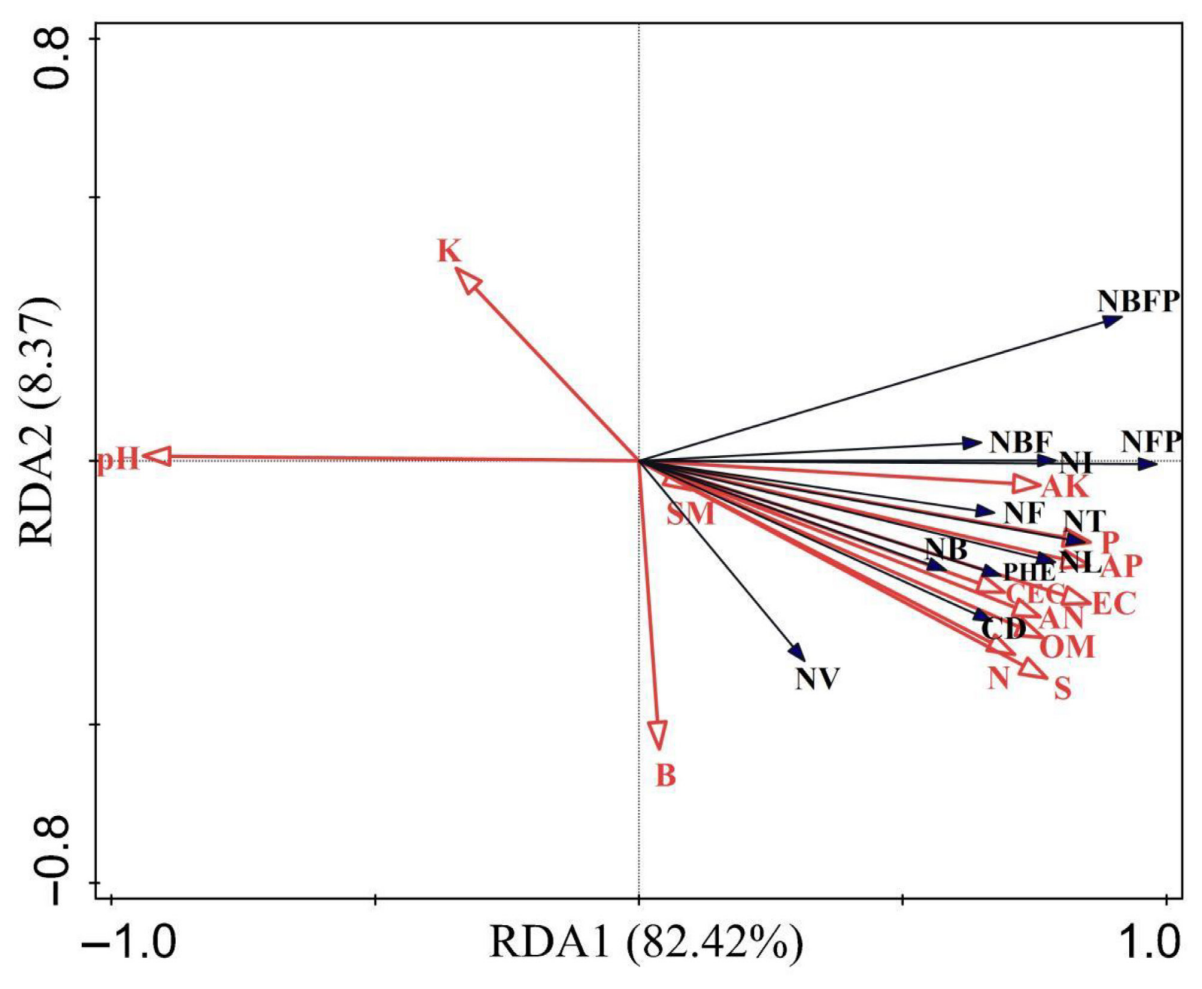
The redundancy analysis (RDA) of growth, flowering characteristics, and soil physicochemical properties of *Salvia daiguii*. The black arrows and font indicate the growth and flowering indicators of the plants (see Table 2 for full abbreviations), and the red arrows and font indicate the soil's physicochemical properties (see Table 3 for full abbreviations). An angle of <90° between the arrows of soil physicochemical properties along with plant growth and flowering indicators with the same direction indicates a positive correlation between soil physicochemical properties along with plant growth and flowering indicators, while the opposite indicates a negative correlation.

In this case, 82.42% and 8.37% of the variance was explained by RDA 1 and RDA 2, respectively, totaling 90.79% of the variance information explained by the two axes. This well explained the relationship between the growth and soil nutrition status of *S. daiguii*. Here, RDA 1 mainly reflected the changes in soil OM, K, SM, AK, AN, and N, with the largest contribution being that of OM at 64.2%; this indicated that soil OM was significantly and positively correlated with plant growth and flowering, i.e, plant growth and flowering indicators increased significantly with increasing soil OM (*p* < .05, Figure 3, Table 4). The RDA 2 axis mainly reflected the change in soil pH, contributing 13.8% of the variance in data. Indicators of plant growth and flowering decreased significantly with increasing soil pH (*p* < .05, Figure 3, Table 4). Thus, OM deficiency and slightly alkaline soils significantly affect the growth and flowering of *S. daiguii*.

**TABLE 4:** Explanation rate of redundancy analysis of soil physicochemical properties on the growth status of *Salvia daiguii*.

Environmental factors	Contribution (%)	Pseudo‐*F*	*p*‐Value
Organic matter (OM)	64.2	12.5	.008
Potential hydrogen (pH)	13.8	3.8	.016
Electrical conductivity (EC)	5.9	1.8	.156
Boron (B)	5.5	2.1	.122
Soil moisture (SM)	3.8	1.7	.210
Sulfur (S)	3.5	2.2	.196
Available nitrogen (AN)	1.9	1.4	.412
Phosphorus (P)	1.3	<0.1	1
Available phosphorus (AP)	<0.1	<0.1	1
Potassium (K)	<0.1	<0.1	1
Available potassium (AK)	<0.1	<0.1	1
Nitrogen (N)	<0.1	<0.1	1
Cation exchange capacity (CEC)	<0.1	<0.1	1

### 3.5 Niche characteristics of *S. daiguii*

#### 3.5.1 Community species composition and importance value characteristics

The four sample plots surveyed in the two populations discussed in Section 2.5 earlier (two plots each at the TNFP and ZEFR populations) were in herbaceous communities. We surveyed a total of 62 plant species from 35 families, of which 13 species had IVs greater than 1% (Table 5). We found that three plants, *Pilea notata*, *Strobilanthes pentstemonoides*, and *Houttuynia cordata*, were present in all four sample plots, where the IVs of *P. notata* and *S. pentstemonoides* were 15.63 and 18.23, respectively, which were greater than that of *S. daiguii* (9.23; Table 5). In addition, *Lycianthes lysimachioides* appeared only in the two TNFP plots, where it had a high IV of 25, with an average of 13.77, also much greater than the IV of *S. daiguii* (Table 5).

**TABLE 5:** Importance values and niche widths of *Salvia daiguii*.

Species	Importance value (%)				Mean ± SE	Niche breadth	
	ZEFR		TNFP			*B*_*i*_	*B*_*s*_
	1	2	3	4			
1. *Salvia daiguii*	10.00	14.76	3.42	8.75	9.23 ± 2.02	3.36	0.84
2. *Pilea notata*	23.87	1.72	14.71	22.21	15.63 ± 4.37	3.05	1.03
3. *Strobilanthes pentstemonoides*	16.66	1.67	29.51	25.07	18.23 ± 5.31	2.99	1.07
4. *Lycianthes lysimachioides*	0.00	0.00	25.57	29.51	13.77 ± 6.92	1.99	0.71
5. *Pieris multifida*	9.65	14.43	0.00	0.00	6.03 ± 3.13	1.92	0.50
6. *Selaginella moellendorffii*	8.34	12.94	0.00	0.00	5.32 ± 2.78	1.92	0.47
7. *Carex thibetica*	5.20	14.61	0.00	0.00	4.95 ± 2.98	1.63	0.43
8. *Houttuynia cordata*	5.67	6.30	1.59	1.01	3.64 ± 1.18	2.82	0.45
9. *Isodon amethystoides*	3.47	9.33	0.00	0.00	3.20 ± 1.91	1.65	0.34
10. *Impatiens piufanensis*	0.00	0.00	3.65	7.29	2.74 ± 1.51	1.80	0.31
11. *Boehmeria nivea*	5.71	1.31	0.92	0.00	1.98 ± 1.10	1.79	0.26
12. *Youngia japonica*	0.00	0.00	3.38	4.21	1.90 ± 0.96	1.97	0.25
13. *Ficus tikoua*	0.83	2.04	1.14	0.00	1.00 ± 0.36	2.63	0.17

#### 3.5.2 Niche width

The Shannon niche widths were in general agreement with the ranking of IVs, 1.03 and 1.07 for *P. notata* and *S. pentstemonoides*, respectively, which were greater than that for *S. daiguii* (0.84), and slightly lower for *L. lysimachioides* (0.71) than that for *S. daiguii* (Table 5). However, Levins's niche widths were generally consistent with the importance ranking, except for *H. cordata* (2.82) and *Ficus tikoua* (2.63). The Levins' niche widths were 3.05 and 2.99 for *P. notata* and *S. pentstemonoides*, respectively; although they were slightly lower than that for *S. daiguii* (3.36), these were essentially the same (Table 5).

#### 3.5.3 Niche overlap

The 13 species with natural community IVs greater than 1% comprised 78 niche overlap values, of which 43 niche overlap values exceeded 0.50, accounting for 55% of the total. In addition, 13 niche overlap values were 0, accounting for about 17% of all niche overlap values (Table 6). The niche overlap values of *S. daiguii* with the dominant plants in the natural community ranged from 0.40 to 0.95. Except for *L. lysimachioide*s and *Youngia japonica*, the niche overlap values of all other dominant plants exceeded 0.6, with *H. cordata* having the highest niche overlap value with *S. daiguii*, which was 0.94, followed by *Selaginella moellendorffii*, *Pieris multifida*, *Carex thibetica*, *Isodon amethystoides*, *F. tikoua*, *P. notata*, *Boehmeria nivea*, and *S. pentstemonoides* (Table 6).

**TABLE 6:** Niche overlap of plant populations of *Salvia daiguii*.

No.	1	2	3	4	5	6	7	8	9	10	11	12
1	—											
2	0.71	—										
3	0.60	0.92	—									
4	0.44	0.74	0.91	—								
5	0.88	0.41	0.25	0.00	—							
6	0.89	0.40	0.25	0.00	1.00	—						
7	0.86	0.27	0.17	0.00	0.97	0.97	—					
8	0.94	0.62	0.48	0.21	0.97	0.96	0.90	—				
9	0.86	0.28	0.17	0.00	0.97	0.98	1.00	0.91	—			
10	0.46	0.74	0.84	0.97	0.00	0.00	0.00	0.19	0.00	—		
11	0.67	0.72	0.50	0.00	0.73	0.71	0.53	0.82	0.54	0.07	—	
12	0.44	0.74	0.90	1.00	0.00	0.00	0.00	0.21	0.00	0.98	0.10	—
13	0.85	0.45	0.49	0.30	0.89	0.87	0.89	0.90	0.89	0.21	0.58	0.29

*Note*: The species numbered 1–13 in this table correspond to those in Table 5.

## 4 DISCUSSION

In the present study, we found 274 individuals on a rock about 200 m from ZEFR1, which is the largest number of sites found to date. Based on previous and current surveys, *S. daiguii* is still only distributed in three populations near Tianmenshan National Forest Park in Zhangjiajie City, TNFP (TNFP1 and TNFP2), TGM, and ZEFR (1 and 2). The number of plants surviving in all populations of *S. daiguii* totaled 500, and the population continued to decline compared to previous surveys, with only 118 mature individuals. This rare species grows mainly on the rocks of cliffs, under cliffs, or on rocks in a streambed. Plant growth, flowering characteristics, and soil nutrients were significantly higher in the ex situ plants protected at SCBG than in the wild populations. The RDA further revealed that OM deficiency and slightly alkaline soils significantly affect the growth and flowering of *S. daiguii*. The results of the niche analysis showed that *S. daiguii* was distributed only in herbaceous/vine communities, where *P. notata*, *S. pentstemonoides*, and *L. lysimachioides* had larger IVs than *S. daiguii*, with niche widths similar to that of *S. daiguii*. The niche overlap values of most of the dominant plants in the natural communities were above 0.6. We concluded that poor environmental conditions, low seedling renewal rate, a lack of soil OM, slightly alkaline soil in the habitat, and niche characteristics of dominant plants in the natural community are the main ecological factors affecting the growth and dispersal of *S. daiguii*.

### 4.1 Distribution and endangered status of *S. daiguii*

Wei et al. (2019) surveys and specimen records found that *S. daiguii* was only distributed in two sites totaling less than 400 (<200 per site) individuals in an 8 km^2^ area of Tianmenshan National Forest Park (Wei et al., 2019; Figure 1‐TNFP 1 and TGM). Xiao, Huang, and Wei (2022) and Xiao, Liu, et al. (2023) conducted extensive surveys in more than 25 provinces and cities during 2011–2021 and also found this species only in the vicinity of Tianmenshan National Forest Park, Zhangjiajie City, Hunan Province, China (Figure 1‐TNFP 2 and ZEFR 1). In the present study, although we found the largest point ZEFR 2 to date, with 274 individuals, it was still restricted to the vicinity of Tianmenshan National Forest Park. Given the location of these sites and the lack of necessary barriers, as a result, *S. daiguii* currently exists in only three populations, TNFP, TGM, and ZEFR (Figure 1). This is a direct indication of the narrow distribution of *S. daiguii*.

The current survey showed that the all population comprises a total of about 500 plants, of which more than 70% of the plants were young individuals, only about 20% were adult breeding individuals, and less than 10% were seedlings. In addition, the size of the TNFP 1 and TGM sites has declined rapidly from <200 individuals per population in 2019 to 15 and 30 individuals per site in 2022. Based on the IUCN classification criteria (IUCN, 2024): the number of mature individuals in all populations is less than 250 individuals (C); continuing population declines have been observed (2); the number of mature individuals is extremely fluctuating (b). Thus, we recommend that *S. daiguii* be classified as Critically Endangered C2b. In addition, *S. daiguii* has a narrow geographical distribution, requires a specific habitat, is subject to long‐term environmental and anthropogenic disturbances, has extremely small populations, and is vulnerable to extinction. Based on these characteristics and the assessment criteria for listing species on China's list of Plant Species with Extremely Small Populations (PSESP) (Ma et al., 2013; Yang et al., 2020), *S. daiguii* is also a PSESP.

### 4.2 Habitat factors facing extinction of *S. daiguii*

The Tianmenshan National Forest Park is located about 20 km from the Wulingyuan Scenic Area, one of the three natural heritage landscapes included in the World Heritage List of the United Nations Educational, Scientific, and Cultural Organization, and it was the first national forest park to be established in China (Yan, 2003). The development of forest tourism and human activities may also have contributed to landscape fragmentation, habitat loss, and abrupt population declines of *S. daiguii* (Clark‐Tapia et al., 2021; Paglia et al., 2022).

Zhangjiajie City is characterized by a landscape with quartz sandstone pillars (Yan, 2003), where *S. daiguii* grows mainly on the rocks of the cliffs, under cliffs, or on rocks in a streambed. Apparently, the habitat conditions for *S. daiguii* are very harsh because habitats on cliffs or rocks have vertically exposed or near‐vertical areas, which are extremely unstable and fragmented habitats. Nevertheless, cliffs and the rocks of streams may affect the survival of species inhabiting such habitats due to the rocky structure, soil erosion, rainfall, and loss of soil water and nutrients (Clark‐Tapia et al., 2021; Larson et al., 2000). These extreme fragmentation and fragile habitats may have contributed to the narrow geographical distribution and endangered status of *S. daiguii*.

*Salvia daiguii* and *S. cavaleriei* have similar morphological characteristics and are sympatric (Wei et al., 2019). In addition, whole plants of *S. cavaleriei* are often used for the treatment of metrorrhagia, hematemesis, and traumatic hemostasis in folklore (Zheng et al., 2013). Therefore, *S. daiguii* is often misidentified as *S. cavaleriei* and may be heavily harvested, which may also contribute to population declines in the former.

Also, Species Distribution Models can simultaneously consider the effects of climate, topography, precipitation, and human activities on a species, as well as predict its potential distribution (Hazarika et al., 2023; Ye et al., 2021). Therefore, Species Distribution Modeling should be taken into consideration in exploring factors affecting the endangerment and conservation of *S. daiguii* in the future.

### 4.3 Effect of soil nutrients on the growth and flowering of *S. daiguii*

The adequacy of nutrients in the soil is an important aspect that can help to ensure plant growth (Osvalde et al., 2021). The present study shows that plant growth, flowering characteristics, and soil physicochemical properties of ex situ plants in SCBG are much superior to those of plants growing in the wild. Further RDA revealed that OM deficiency and slightly alkaline soils significantly affect the growth and flowering of *S. daiguii*. Compared to the SCBG plants, the plants in the wild grow mainly on cliffs or rocks. However, the rocky structure of the habitat may be low in OM, which may cause a loss of soil nutrients, which greatly affects the rate of decomposition of OM (Ardestani & Ghahfarrokhi, 2021; Luo et al., 2021). Soil pH was negatively correlated with plant growth and flowering indicators for *S. daiguii*. The soils of the ex situ plants at SCBG were neutral, while the soils in the wild habitat were slightly alkaline, which indicated that the *S. daiguii* favored a growth environment with neutral soils. In conclusion, the slightly alkaline soil of the natural habitat and the lack of soil OM may be two of the main factors limiting the growth, flowering, and geographic distribution of *S. daiguii*.

### 4.4 Niche characteristics of *S. daiguii*

*Salvia daiguii* occurs in habitats with a high level of biodiversity with a rich community of species dominated by herbs. In our study, *S. daiguii* had a large niche width and was still very adaptive and competitive in its habitat and community. However, the plant abundance, height, density, and cover of *P. notata* and *S. pentstemonoides* were much higher than those of *S. daiguii*; their niche IVs competed with those of *S. daiguii*, and these co‐occurring species affect the growth and reproduction of *S. daiguii*. In addition, most of the dominant species in the community had niche overlap values greater than 0.6, which may be the result of habitat loss for *S. daiguii*, resulting in habitat occupation by these species that prefer moist environments and slightly alkaline soils. Notably, although the niche overlap value of *L. lysimachioides* was lower than 0.5, its IV and niche width were similar to those of *S. daiguii*; so, it may also pose a threat to the growth and reproduction of *S. daiguii* in the future.

## 5 CONSERVATION RECOMMENDATIONS FOR *S. DAIGUII*

In general, *S. daiguii* has a narrow geographical distribution, requires special habitat types, is subject to long‐term environmental and anthropogenic disturbances, and experiences a lack of soil nutrients in addition to competition for niches from co‐occurring species in the community, resulting in extremely small populations, creating an urgent need for conservation. Therefore, we propose the following conservation recommendations: (1) Based on the IUCN classification criteria and the definition and characteristics of PSESP, it is recommended that *S. daiguii* be classified as Critically Endangered C2b and PSESP. (2) Accelerate the establishment of nature reserves to protect these threatened individuals by strictly prohibiting the harvesting of these plants by any reasonable means necessary. (3) The existing population is distributed in the Tianmenshan National Forest Park and its nearby areas. The importance and significance of the conservation of endangered plants should be promoted among the public and villagers in association with tourism management areas. (4) About 500 plants have been propagated at SCBG by tissue culture, division, and sowing from 2011 to 2021, and about 1000 plants have been propagated at Nanjing Forestry University by sowing seedlings in 2022. We propose reintroducing propagated plants to the field to increase the number of field populations according to the principle of reintroduction to increase the number of field populations. (5) *Salvia daiguii* is exposed to causative factors in population ecology, but the causative factors of this plant in pollination biology, seed physiology ecology, and genomics are still unclear. Future strengthening of research is suggested on these aspects, and comprehensive conservation recommendations should be made for the reasonable development and use of these plant resources.

## AUTHOR CONTRIBUTIONS

**Han‐Wen Xiao:** Conceptualization (equal); data curation (equal); formal analysis (equal); funding acquisition (equal); investigation (equal); methodology (equal); resources (equal); software (equal); visualization (equal); writing – original draft (equal); writing – review and editing (equal). **Qing‐Shan Liu:** Investigation (equal); methodology (equal). **Yan‐Bo Huang:** Investigation (equal); resources (equal). **Yan Li:** Data curation (equal); formal analysis (equal). **Yu‐Kun Wei:** Conceptualization (equal); methodology (equal); resources (equal); validation (equal); visualization (equal); writing – review and editing (equal). **Ru‐Nan Tian:** Funding acquisition (lead); methodology (equal); project administration (lead); supervision (lead); validation (lead); visualization (lead); writing – review and editing (lead).

## FUNDING INFORMATION

This work was supported by the Chenshan Special Foundations from the Shanghai Municipal Administration of Forestation and City Appearances (No. G222402), A Project Funded by the Priority Academic Program Development of Jiangsu Higher Education Institutions (PAPD), and Postgraduate Research & Practice Innovation Program of Jiangsu Province (KYCX23_1257).

## CONFLIC OF INTEREST STATEMENT

The authors declare that there are no conflicts of interest.

## Data Availability

The data are available at the Dryad Digital Repository: https://datadryad.org/stash/share/uaNieS7pTtgblZgd8CjFJggUYkMIb9‐K7T_7OWebxzY.
